# Risky decision-making after exposure to a food-choice task in excess weight adolescents: Relationships with reward-related impulsivity and hunger

**DOI:** 10.1371/journal.pone.0202994

**Published:** 2018-08-24

**Authors:** María Moreno-Padilla, María José Fernández-Serrano, Gustavo A. Reyes del Paso

**Affiliations:** Department of Psychology, Universidad de Jaén, Jaén, Spain; Technion Israel Institute of Technology, ISRAEL

## Abstract

**Objective:**

To assess the effects of exposure to a food-choice task (appetizing *versus* healthy food) on risky decision-making by excess *versus* normal weight adolescents. We also analyzed the influence of food visualization on hunger levels, as well as group differences in food choices and impulsivity.

**Methods:**

Fifty-six adolescents (aged 13–18 years) classified as excess (n = 27) or normal (n = 29) weight participated in the study. Risky-decision-making was assessed through the Balloon Analogue Risk Task, which was administered before and after a food-choice task. We also evaluated impulsivity traits through the UPPS-P Scale, and subjective hunger levels with a visual analogue scale.

**Results:**

Adolescents with excess weight showed enhanced risky decision-making after the food-choice task compared to normal weight adolescents, as well as increased hunger levels. Furthermore, excess weight adolescents made more appetizing choices, and showed greater scores for Positive Urgency and Sensation Seeking. Reward-related impulsivity measures were positively associated with the number of appetizing choices in the food-choice task. Several associations were found between impulsivity measures, hunger levels and risk-taking variables.

**Conclusions:**

Excess weight adolescents increased their risky-decision-making after food exposure and this augmentation was associated with the increase in hunger levels. Increased hunger levels and risk-taking after food exposure could lead to overeating. Alterations in decision-making caused by food signals may be a long-term risk factor for the development of obesity in adulthood. In modern societies, with the high availability and continuous exposure to food cues, decision-making may be a crucial factor in maintain healthy eating habits in adolescents.

## Introduction

The prevalence of overweight and obesity in adolescence has increased considerably in recent decades [1,2] and excess weight in adolescents is a strong predictor of adult obesity [3]. Overweight and obesity are currently the fifth-leading mortality risk factor and it is associated with increased incidence rates of diabetes, cardiovascular diseases and some types of cancer (World Health Organization [WHO]) [4].

In the last few decades, drastic changes in the environment and lifestyles have modified the way we perceive foods and regulate their intake [5]. The availability of a wide range of foods, and overexposure to marketing-related images of foods in Western societies, has led to what, and how much, to eat becoming a decision-making matter. Obesity has been proposed as a problem of food addiction, with overeating being explained as an imbalance between motivational and control-inhibition systems [6,7]. From this theory, it is proposed that in vulnerable individuals, the consumption of large amounts of appetizing food (high in fat and/or sugar) could cause an imbalance in the interaction of these systems, resulting in an increase in the motivational-reinforcing value of appetizing food and a weakening of the control-inhibitory system [7]. This deficit in control and inhibitory influences would lead to impulsive and compulsive intake of appetizing foods, and as a consequence, to the development and maintenance of obesity [7].

The impact of inhibitory control on eating behaviour seems to be particularly relevant during adolescence [8], a developmental period in which both motivational tendencies and impulse control skills strongly modulate goal-directed behaviour [9]. Furthermore, decision-making skills are particularly relevant in adolescents, in whom executive control areas (prefrontal areas) are not completely developed and seem to maximize reward at the expense of risk [9].

A relevant perspective in impulsive decision-making is the concept of risk-taking. Risk-taking propensity refers to the appetitive processes underlying a behavioral predisposition to take risks in response to signals for potential reward, which also confers a probability of aversive results [10]. In recent years, the concept of risk-taking has been used to describe impulsive behavior in drug addiction and obesity [11,12]. Previous studies have repeatedly shown that drug abusers are risk prone, as evidenced by self-reports of sensation seeking [13] and responses to laboratory risk-taking tasks [14]. Obesity is also associated with greater risk-taking, showing an association with risky patterns of responses to tasks like the Iowa Gambling Task (IGT) [15].

Adolescents are known to have a tendency to take more and greater risks than individuals of other age ranges in many life domains, such as unprotected sex, criminal behavior, dangerous driving, and experimenting with alcohol and other drugs [16]. Furthermore, adolescents who are reward sensitive and have difficulties in controlling their behavior appear to be most susceptible to involvement in risky behavior [17].

There is increasing evidence that individual differences in the tendency to overeat are related to impulsivity, possibly due to increasing reactivity to environmental food-related cues [18]. Neurocognitive studies have shown that obesity and addiction are both associated with increased impulsive decision-making and attentional bias in response to drug or food cues, respectively [19]. Several studies have analyzed attentional bias in individuals with obesity, but the results have been inconsistent [20,21]. When participants were tested in a hungry state, no differences were found, but when they were satiated at the time of testing, greater attentional bias was found in excess compared to normal weight adults [22]. However, specific literature on adolescents is scarce. To the best of our knowledge, only one study has found increased attentional bias and impulsivity to food cues in adolescent girls, as well as reduced activation of frontal inhibitory regions [23].

Regarding appetitive motivation, substance use disorders (addiction) and obesity, as well as subjective states of craving and hunger, are associated with attentional bias for drug- and food-related stimuli, respectively [24,25]. Furthermore, previous studies showed that drug-cue reactivity is positively associated with increases in impulsivity and risk-taking in substance abusers [26].

Decision making in eating behaviour can be studied by food choices tasks. Food decisions concern what, when, and how much to eat. Food choices can lead to overconsumption, when there is an increased preference for appetizing food (high in fat and/or sugar). Therefore, the study of decision making is extremely important in this population, since decision making based in unhealthy choices can lead to weight more gain and develop or maintain obesity.

This study examined the effect of exposure to food pictures, in a food-choice task, on subsequent measures of risky decision-making and hunger levels in adolescents with excess *versus* normal weight. Risk taking was assessed through the Balloon Analogue Risk Task (BART). Riskiness on the BART was related to self-reported engagement in real-world risk-taking behaviours [27]. To study the influence of exposure to food pictures, performance on the BART and feelings of hunger were evaluated both before and after the food choice task. Impulsivity traits were also evaluated with the UPPS-P Scale. This questionnaire measures five components of impulsive behavior: Sensation Seeking, Positive Urgency, Negative Urgency, Lack of Premeditation and Lack of Perseverance. We hypothesized that adolescents with excess weight would show greater risky decision-making after exposure to food pictures, while no change was expected in normal weight adolescents. We expected to find greater increases in hunger levels after the food-choice task in excess *versus* normal weight participants and more food-appetizing choices among excess weight adolescents. Finally, concerning relationships among variables, we expected to find positive associations between impulsivity traits related to reward seeking (as Positive Urgency and Sensation Seeking), risk-taking outcomes, hunger levels and number of appetizing choices.

## Methods

### Participants

In total, 56 adolescents (24 males and 32 females) aged between 13 and 18 years participated in the study. They were selected based on their age adjusted body mass index (BMI) percentile in accordance with the guidelines of the International Obesity Task Force [28] criteria: normal weight participants (n = 29), with age-adjusted BMI values in the range between the 5^th^ and the 84^th^ percentile, and excess weight participants (n = 27), with age adjusted BMI values above the 85^th^ percentile. Although our inclusion criteria for the normal weight group allowed for people with BMIs up to the 84th percentile, the maximum BMI of any participant in the normal weight group was in the 70th percentile. Socio-demographic, BMI, waist-hip ratio and fat percentage data are displayed in Table 1. Participants were recruited from high schools located in Jaén (Spain). The inclusion criteria were: (i) aged between 13 and 18 years; and (ii) no history of neurological or psychiatric disorders. All participants had normal or corrected-to-normal vision.

**Table 1:** Participants’ socio-demographic characteristics, BMI and percentage of body fat.

	Excess weight		Normal weight		t^a^/chi square^b^		p
	Mean	SD	Mean	SD			
Age	15.28	1.82	15.43	1.39	-0.36^a^		0.723
Sex (%Men/women)	46.7/53.3		36.7/63.3		1.72^b^	0.189	
BMI	28.33	2.74	20.12	2.05	12.76^a^		<0.001
% Body fat	27.34	7.71	17.90	7.16	4.75^a^		<0.001

^a^value of Student’s t

^b^value of Chi-square χ2

### Instruments

#### Self-reported measures

Spanish version of the short UPPS-P impulsive behavior scale [29]: the UPPS-P is a 20-item inventory designed to measure five components of impulsive behavior: sensation seeking, lack of perseverance, lack of premeditation and urgency (positive and negative). Each item on the UPPS-P is rated on a four-point scale ranging from 1 (strongly agree) to 4 (strongly disagree). Positive Urgency is defined as the tendency to act rashly to obtain reinforcement when experiencing positive emotions, while Negative Urgency refers to the tendency to engage in impulsive behaviours under conditions of negative affect. Sensation seeking describes individuals' tendency to seek out novel, complex, and intense sensations and experiences, and a predisposition to take risks to realise these experiences. Lack of Premeditation refers to the tendency to think and reflect on the consequences of an act before engaging in that act or taking a decision. Lack of Perseverance refers to an individual’s inability to remain focused on a task that may be boring or difficult.

- A visual analogue scale (VAS) designed to rate hunger levels. Participants had to indicate how hungry they were feeling on a scale ranging from 1 to 10 (not hungry to very hungry).

#### Risk-taking task

The BART [30] is a 20-trial computerized task that models real-world risk behavior according to the concept of balancing the potential for reward and harm. The participant is presented with a balloon and asked to pump it up by clicking a button on the screen. With each pump, the participant obtains 25 cents and the balloon increases slightly in size. However, each balloon also has a concealed probability of exploding after an unspecified number of pumps. Participants were told that at some point each balloon would burst. Before the balloon explodes, the participant can press “Collect money,” which saves his or her earnings to a permanent bank. If the balloon explodes before the participant collects the money, all earnings for that balloon are lost, and the next balloon is presented. Each successful click increases the participants’ temporary payoff but increases the risk of the balloon exploding. Thus, each pump confers not only greater risk but also greater potential reward.

In this version of the task, the maximum number of pumps possible for a given balloon was 128, thus the probability of the balloon exploding on Pump 1 was 1/128. If there was no explosion after this first pump, the probability of explosion on Pump 2 was 1/127, and so on up until the 128th pump. Accordingly, the average break point or “optimal stopping point” for each balloon was 64 pumps.

Dependent variables are the average number of pumps of unexploded balloons,the number of exploded balloons (higher scores indicate greater risk-taking propensity) and the amount of money obtained.

#### Food-choice task

A food preference decision-making task was used in this study. Two types of food pictures were utilized: appetizing (high levels of fats and/or sugars) and healthy. Appetizing cues included, for instance, sausages and chocolate and healthy cues included, for instance, fruits and salads. In each trial, pairs of pictures of these different types of foods were presented in three conditions (appetizing vs. healthy, appetizing vs. appetizing and healthy vs. healthy). Participants had to choose between the two options by pressing a computer keyboard. The total duration of the task was about 5 minutes. Each trial begins with a fixation cross which lasts from 3 to 6 seconds, varying between trials. Then, the images of the two options appear for 5 seconds (one on the left side of the screen and the other one on the right side of screen, with the positions of the appetizing and healthy foods varying among trials). The order of presentation of the images was counterbalanced across the participants. Then, the fixation cross was represented. There were a total of 30 choice trials, with 10 choices for each decision category, preceded by four practice trials. The outcome measure was the number of selections of each type of food.

### Procedure

Height, weight and body composition measures (Bodystat®1500 monitoring unit) were collected on arrival of the participant. Subsequently, the UPPS-P impulsivity questionnaire was administered followed by the BART (pre-task). Then, participants performed the food-choice task, and immediately after, the BART was administrated again (post-task). Subjective hunger evaluation (VAS) was carried out before (and after UPPS-P) (pre-task) and after completion of the food-choice task (post-task). The Ethics Committee for Human Research of the Universidad de Jaén approved the study. Both participants and parents signed informed consent forms.

### Statistical analyses

Group comparisons were carried out with Student *t*-test for independent samples. BART and hunger measures were analyzed by repeated measures ANOVA with Time (pre- and post-task) as the repeated-measures factor and Group (Excess vs. Normal weight) as the between-subject factor. Additionally, we have carried out ANCOVA analyses including the covariables of Sensation Seeking and Positive Urgency in order to assess the influence of these variables on the pre- to post- change. The computation of planned á priori correlations between variables were performed by Pearson correlations. Finally, mediation analyses were performed with the PROCESS macro for SPSS. To assess the significance of partial mediation effects, confidence intervals from the bootstrapping estimation techniques were used. For a significant meditational effect, the limits of confidence interval should not include the 0 value [31,32]. In order to simplify these analyses, change scores were calculated as the post-task value minus the pre-task value.

## Results

### Self-reported measures

The groups differed in two dimensions of the impulsivity questionnaire (UPPS-P), sensation seeking (t = 2.17, p = 0.034, δ = 0.58) and positive urgency (t = 2.14, p = 0.037, δ = 0.56), with greater scores in the excess *versus* normal weight adolescents (Table 2). A Time x Group interaction was found for hunger VAS scores (F_1,54_ = 8.56, p = 0.005, $\eta_{p}^{2}$ = 0.14). Although both groups showed significant increased hunger levels, the increase from the pre- to post-task evaluation was greater in excess weight (F_1,26_ = 33.72, p<0.001, $\eta_{p}^{2}$ = 0.57) *versus* normal weight adolescents (F_1,28_ = 17.37, p< 0.001, $\eta_{p}^{2}$ = 0.38) (Table 2).

**Table 2:** Means and standard deviations (SD) of impulsivity (UPPS-P), hunger (VAS) measures, appetizing and healthy choices. Results of the group comparisons (t and p) are also displayed.

	Excess weight		Normal weight		t	p
	Mean	SD	Mean	SD		
UPPS-P Urg-	11.89	8.65	10.90	3.44	0.57	0.570
UPPS-P Urg+	9.96	2.12	8.86	1.73	2.14	0.037
UPPS-P SS	11.19	2.63	9.62	2.74	2.17	0.034
UPPS-P LPrem	8.37	1.94	8.24	2.59	0.21	0.835
UPPS-P LPers	7.63	2.10	8.31	2.56	-1.08	0.284
UPPS-P Total	46.04	9.63	47.03	8.09	-0.42	0.676
Hunger Pre Hunger Post	1.35 4.94	1.48 2.62	2.35 3.88	2.26 2.77	-1.94 1.48	0.057 0.144
A_Choices H_Choices	7.11 2.89	2.06 2.06	4.38 5.62	2.53 2.53	4.41 -4.41	<0.001 <0.001

*Note*: Urg-: Negative Urgency; Urg+: Positive Urgency; SS: Sensation Seeking; LPrem: Lack of Premeditation; LPers: Lack of Perseverance; A_Choices: appetizing choices; H_Choices: healthy choices

Additionally, results of ANCOVA showed that Sensation Seeking had a significant effect on the change in hunger levels in the whole sample (F_1,53_ = 4.13, p = 0.047, $\eta_{p}^{2}$ = 0.072). Furthermore, a Sensation Seeking x group x time (F_1,53_ = 8.40, p = 0.001, $\eta_{p}^{2}$ = 0.241) interaction was found. In order to analyze this interaction we explored the effect of the covariable in each group separately. Sensation Seeking influenced the change in hunger levels in the excess weight group (F_1,25_ = 7.97, p = 0.009, $\eta_{p}^{2}$ = 0.242), but not in the normal weight group (F_1,27_ = 0.03, p = 0.869, $\eta_{p}^{2}$ = 0.001). Regarding food choices, excess weight adolescents chose significantly more appetizing foods than normal weight adolescents (Table 2).

### Risk-taking task (BART)

**Fig 1:**
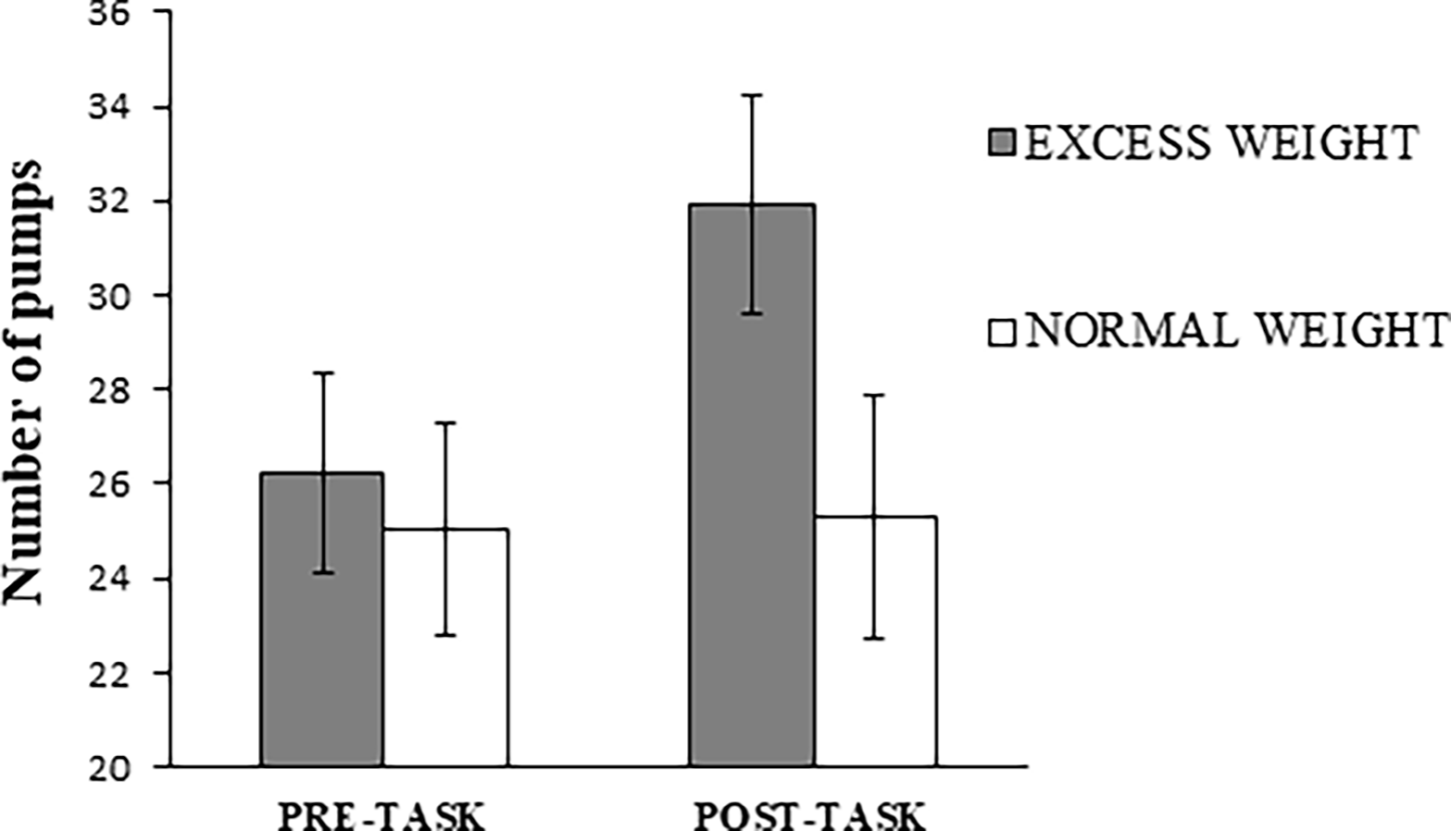
Average number of pumps on unexploded balloons in the pre-task and post-task evaluations as a function of group.

**Fig 2:**
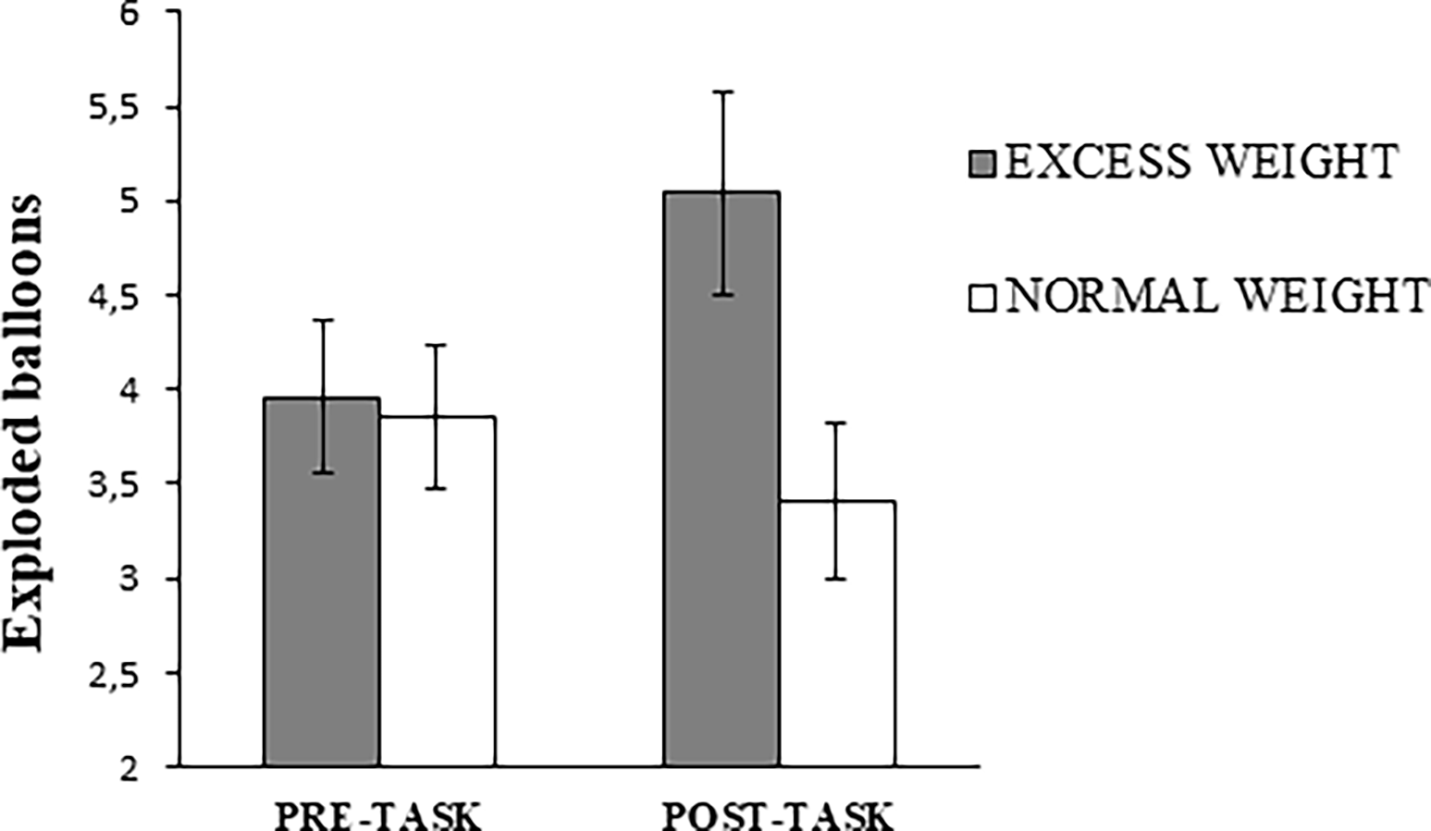
Number of exploded balloons in the pre-task and post-task evaluations as a function of group.

Significant Time x Group interactions were found for the average number of pumps of unexploded balloons (F_1,54_ = 5.68, p = 0.021, $\eta_{p}^{2}$ = 0.10) (Fig 1) and exploded balloons (F_1,54_ = 7.38, p = 0.009, $\eta_{p}^{2}$ = 0.12) (Fig 2). The average number of pumps of unexploded balloons (F_1,26_ = 14.57, p = 0.001, $\eta_{p}^{2}$ = 0.36) and exploded balloons (F_1,26_ = 6.33, p = 0.018, $\eta_{p}^{2}$ = 0.20) increased in excess weight adolescents after the food-choice task, while no significant changes were observed in normal weight adolescents (p>0.23). No Time x Group interaction was found for the amount of money obtained (F_1,54_ = 2.34, p = 0.132, $\eta_{p}^{2}$ = 0.04). While no group differences in the BART were observed in the pre-task evaluation (exploded balloons: p = 0.855; average number of pumps on unexploded balloons: p = 0.702), the above-described differential responses to the food choice task led to increased risk-taking (exploded balloons) in the excess *versus* normal weight adolescents during the post-task evaluation (t = 2.43;p = 0.019; δ = 0.67). No differences were found between groups in the money obtained before (t = 0.10, p = 0.917, δ = 0.03) and after (t = 1.39, p = 0.172, δ = 0.37) food visualization.

The inclusion of Sensation Seeking and Positive Urgency as covariables in these analyses did not change the above results.

### Associations between measures

In the whole sample (Table 3), the change in hunger levels was positively associated with the change in the number of exploded balloons, the number of exploded balloons after the food-choice task and the average number of pumps on unexploded balloons before and after the food-choice task. Sensation Seeking was positively associated with the change in hunger levels, the number of appetizing choices made in the food choice task, the number of exploded balloons and the average number of pumps on unexploded balloons after the food-choice task. Positive Urgency and UPPS-P total scores were positively correlated with the number of appetizing choices. Finally, BMI was positively associated with the change in the number of exploded balloons, the number of exploded balloons after the food-choice task, Positive Urgency, Sensation Seeking and the number of appetizing choices.

**Table 3:** Pearson correlations between variables in the whole sample.

*n* = 56	Hunger_ Change	UPPSP_ T	UPPSP_ U+	UPPSP_ SS	BMI
Hunger_change	1	0.05	0.03	0.34*	0.25
EB_Change	0.33*	-0.13	-0.08	0.23	0.30*
ANPUB_Change	0.08	-0.20	0.09	0.17	0.18
EB_PRE	0.20	0.16	0.06	0.22	0.09
EB_POST	0.43**	0.01	-0.02	0.37**	0.33*
ANPUB_PRE	0.28*	0.14	-0.06	0.18	0.11
ANPUB_POST	0.34*	-0.06	0.02	0.28*	0.22
A_Choices	0.24	0.42**	0.36**	0.27*	0.45**
BMI	0.25	0.04	0.33*	0.27*	1

EB: Exploited Balloons; ANPUB: Average Number of Pumps on Unexploded Balloons

T: total; U+: Positive Urgency; SS: Sensation Seeking; A_Choices: appetizing choices; BMI: Body Mass Index

** Correlation is significant at the 0.01 level (2-tailed)

* Correlation is significant at the 0.05 level (2-tailed)

In the excess weight group (Table 4), the change in hunger levels was positively associated with the change in the number of exploded balloons, the number of exploded balloons after the food-choice task, the average number of pumps on unexploded balloons before and after the food-choice task and sensation seeking scores. Finally, Sensation Seeking was positively associated with the number of exploded balloons after the food choice task and UPPS-P total scores were positively correlated with the number of appetizing choices. In the normal weight group (Table 5), UPPS-P Total scores correlated positively with the number of appetizing choices.

**Table 4:** Pearson correlations between variables in excess weight group.

*n* = 27 Excess weight	Hunger_ Change	UPPSP_ T	UPPSP_ U+	UPPSP_ SS	BMI
Hunger_change	1	0.14	-0.11	0.49**	-0.30
EB_Change	0.46*	0.03	-0.16	0.16	0.06
ANPUB_Change	0.14	-0.19	0.14	-0.17	-0.14
EB_PRE	0.32	0.07	0.08	0.38	-0.01
EB_POST	0.60**	0.07	-0.07	0.41*	0.04
ANPUB_PRE	0.43*	0.26	-0.01	0.38	-0.01
ANPUB_POST	0.48**	0.02	0.08	0.27	-0.15
A_Choices	0.24	0.57**	0.20	0.23	0.22
BMI	-0.30	0.12	0.22	0.01	1

EB: Exploited Balloons; ANPUB: Average Number of Pumps on Unexploded Balloons

T: total; U+: Positive Urgency; SS: Sensation Seeking; A_Choices: appetizing choices; BMI: Body Mass Index

** Correlation is significant at the 0.01 level (2-tailed)

* Correlation is significant at the 0.05 level (2-tailed)

**Table 5:** Pearson correlations between variables in normal weight group.

*n* = 29 Normal weight	Hunger_ Change	UPPSP_ T	UPPSP_ U+	UPPSP_ SS	BMI
Hunger_change	1	-0.04	-0.03	-0.03	0.14
EB_Change	-0.14	-0.30	-0.25	0.13	-0.07
ANPUB_Change	-0.27	-0.19	-0.14	0.29	-0.24
EB_PRE	0.03	0.27	0.04	0.07	0.32
EB_POST	-0.10	-0.02	-0.19	0.19	0.24
ANPUB_PRE	0.10	0.04	-0.14	0.01	0.27
ANPUB_POST	-0.04	-0.11	-0.21	0.18	0.06
A_Choices	-0.15	0.51**	0.32	0.10	-0.18
BMI	0.14	0.26	0.12	0.12	1

EB: Exploited Balloons; ANPUB: Average Number of Pumps on Unexploded Balloons

T: total; U+: Positive Urgency; SS: Sensation Seeking; A_Choices: appetizing choices; BMI: Body Mass Index

** Correlation is significant at the 0.01 level (2-tailed)

We have considered performing an alpha adjustment for multiple comparisons. However, this would substantially reduce the power of the tests, i.e. increase the chance of Type II errors and reduce the probability of detecting any effects present. Certainly, some of our correlations would not survive correction for multiple testing.

Influential authors like Rothman (1990, 2014) [33,34] claimed that correction for multiple testing may be an essentially wrong option and a research misconception in our field. If one is analyzing psycho-physiological data that are replete with actual associations, the premise for traditional adjustments is difficult to defend. As stated above, adjustments for multiple comparisons may reduce Type I errors, but they do so at the expense of increasing Type II errors.

Results of mediation analysis showed that the change in hunger levels mediated the difference in the number of exploded balloons between the pre- and post- administrations of the BART in excess weight participants (Bootstrapping Lower Limit Confidence Interval = 0.04, Bootstrapping Upper Limit Confidence Interval = 0.54), and not in normal weight participants (Bootstrapping Lower Limit Confidence Interval = -0.14, Bootstrapping Upper Limit Confidence Interval = 0.06).

## Discussion

Results showed that after the food-choice task adolescents with excess weight displayed increased values in the two risk-taking measures of the BART than adolescents with normal weight. Adolescents with excess weight also showed a greater increase in hunger levels (VAS scores) after exposure to the food-choice task. Furthermore, excess weight adolescents showed greater scores in Positive Urgency and Sensation Seeking (UPPS-P), as well as an increased number of appetizing selections in the food-choice task, compared to normal weight adolescents. Finally, significant associations were found between the change in hunger feelings, the number of appetizing choices, risk-taking and reward-related impulsivity measures.

### Food-visualization effects on risk-taking

Our findings suggest that adolescents with excess weight have enhanced reactivity to food cues, since the food-choice task led to an increase in risk-taking in these individuals. Yeomans and Brace (2015) [35] showed similar results in a study comparing restrained *versus* overeating-susceptible healthy women selected according to their scores in on the disinhibition and restraint scales of the Three Factor Eating Questionnaire (TFEQ) [36]. They found that exposure to food cues led to a greater risk propensity (measured with the BART) in women susceptible to overeating in comparison with restrained women. However, they found group differences in BART measures both before and after food cue-exposure, while we only observed differences after the food-choice task. The pre-task discrepancy may be due to differences in the studies samples, as they selected their sample based on uncontrolled eating (TFEQ), while we selected ours based on BMI.

To our knowledge, this is the first study to analyse the influence of food cues visualization on risk-taking in adolescents with excess weight. The fact that excess weight adolescents increase risky decision-making after food exposure may be relevant to our understanding of the role of food cues in the development of unhealthy eating behaviours in modern societies. Motivational mechanisms could be involved in food cue-enhanced risky decision-making. In general, it is known that positive mood states induce increased risk-taking [37], which in turn promotes further gratification-seeking behaviour to maintain a positive mood. For example, undergraduate college students are more likely to drink on days of celebration than during the week [38], and individuals may also engage in risky drinking to enhance a pre-existing positive mood [39]. This hypothesis is in line with our results of greater positive urgency (reward seeking under a positive mood) in excess weight adolescents. In line with this result, Fernández-Serrano et al. (2011) [40], using the Iowa gambling task (IGT) in polysubstance users, found that drug-users (who showed a risky pattern of decision-making under normal conditions) decreased their risk-taking in a negative affective context (visualization of negative images while performing the IGT) to a level similar to that observed in controls, while they increased their risky decisions on the IGT during a positive affective context evoked by drug cue visualization.

### Food-visualization effects on hunger levels and its associations with risk-taking

The food-choice task led to a greater increase in hunger feelings in excess weight group. Food cues could be associated with greater reward value in adolescents with excess weight than in normal weight adolescents. Evidence points to greater neural reactivity in the reward system in obese *versus* normal weight individuals during high-calorie food visualization [41]. Therefore, appetizing food cues may evoke an approach response to reward, leading to greater hunger feelings and enhanced impulsive risk-taking behaviours [42]. In fact, in our study, a greater number of appetizing choices in the food-choice task were found in excess *versus* normal weight adolescents. Therefore, this purported greater underlying reactivity to appetizing food cues in obese individuals may mediate the observed impulsive behaviour after food visualization.

Furthermore, we found a positive association between risk-taking (greater number of exploded balloons and average number of pumps on unexploded balloons) and the change in hunger levels in the whole sample and in the excess weight group particularly, but not in the normal weight group. Besides, the change in hunger levels was also positively associated with the average number of pumps of unexploded balloons after the food-choice task in adolescents with excess weight. Therefore, food visualization and the consequent increase in hunger lead to enhanced risk-taking in excess weight adolescents, what may cause alterations in impulses control and hinder the intake control. Furthermore, subjective hunger may predispose an individual to believe that his/her body is in a state of homeostatic imbalance that must be restored through the intake of food. This may increase the predisposition to overeat in current society, given the ubiquitousness of full of fatty/sweet food cues. Therefore, these results suggest that greater hunger feelings may predispose to enhanced risk-taking in excess weight adolescents, which can lead to greater seeking of the reward consumption, in this case appetizing foods high in fats and / or sugars.

### Reward-related impulsivity measures and its associations with hunger levels and appetizing choices

Excess weight adolescents showed greater scores in Positive Urgency and Sensation Seeking than normal weight adolescents. Available evidence concerning impulsivity traits in obese adolescents is scarce. In the two studies available on adolescents with excess weight, no group differences in impulsivity were found [8, 43]. As a possible explanation for these differences *versus* the current study, the mean age of the excess weight participants in these previous studies was 14.19±1.38 and 14.22±1.4 years, lower than that in our study (15.28±1.82 years). The mean age of our study is characterized by greater freedom and less control by parents, so it is more likely that adolescents around this age develop behaviours such as searching for new experiences or immediate rewards. For example, significant relationships between sensation seeking and adolescent alcohol use, cigarette smoking and marijuana use have been reported, with older adolescents being more likely to engage in these types of risky behaviours [44]. Conversely, Nazarboland and Fath (2015) [45] found greater Sensation Seeking in highly obese adolescents (BMI>35) than in normal weight adolescents.

Sensation Seeking was positively associated with the change in hunger levels in excess weight participants. Furthermore, this variable mediated the difference in hunger levels in this group between the pre- and post- food-choice task evaluations. This suggests that the impulsivity trait may not only be associated with eating preferences, but also with changes in subjective feelings of hunger, which could stimulate overeating and the intake of high-calorie foods, leading to obesity.

Regarding to this, Sensation Seeking has long been associated with elevated drug intake in humans [46]. Therefore, the influence of Sensation Seeking on a greater increase in hunger feelings in excess weight adolescents may support the hypothesis of a greater reactivity to food signals (i.e., increased seeking for rewards and positive reinforcement) in these participants.

A preference for appetizing food in the food-choice task was associated with impulsivity measures in our whole sample. Specifically, Sensation Seeking and Positive Urgency (both related to greater reward sensitivity), which may indicate a mediational role of impulsivity in determining food preferences. These results corroborate previous evidence. Davis et al. (2007) [47] found in women ranging from normal weight to obese that reward sensitivity was positively linked to overeating and high sugar-fat food preferences. Nederkoorn et al. (2010) [48] found that participants with greater impulsivity gained more weight during a 1-year period. It has been proposed that impulsivity may accelerate the acquisition of Pavlovian conditioning to appetitive cues [49]. All of this evidence suggests that exposure to appetitive food cues, via interaction with impulsivity traits, may play an important role in the development of unhealthy eating behaviours. In modern societies, given the high availability of, and frequent exposure to, high calorie foods, individuals with high reward sensitivity are predisposed to consumption beyond their caloric needs. The enhanced preference for fat-sweet foods is explained by their greater reinforcing value, especially in individuals with excess weight [50].

As the incentive salience of appetizing food cues increases, seeking out and consuming this type of food becomes an important goal, exceeding feeding homeostatic regulation [51]. This represents a risky behaviour, since consuming foods high in fat and/or sugar is associated with weight gain in children and adolescents and, therefore, increased risk of obesity [52]. Macchi, MacKew and Davis (2017) [53] assessed eating habits and risk-taking (BART) in adolescents and found that choices on the BART were riskier in adolescents who made unhealthier food choices. These findings are congruent with studies observing that adolescents with higher risk-taking on the BART consistently engaged in greater risk-taking activities outside of the laboratory, such as smoking, drinking, gambling or substance abuse [54–56].

### Limitations

Regarding limitations of the study, there are a number of issues that need to be addressed in future studies, like differentiating among obese, overweight and normal weight participants, the inclusion of objective eating behaviour measures (in the home and/or the lab), and the fulfilment of a more exhaustive decision-making evaluation. In addition, we performed several correlations, which may increase Type I errors. Consequently, results concerning associations between variables should be taken with caution and considered preliminary. Furthermore, future studies should evaluate the longitudinal influence of risk-taking on weight gain. Finally, the lack of a control group not exposed to the food-choice task manipulation makes it difficult to discern whether the changes in hunger feelings and decision-making are due to the visualization of food, the mere passage of time or the repeated administration of the test. Future studies will be necessary to address this limitation by including an appropriate control group.

### Conclusions

In summary, the results showed that excess weight adolescents increased their risky decision-making after food-choice task exposure, where this was associated with an increase in hunger levels. Excess weight in adolescence is a risk factor for the development of future health problems and obesity. In current western societies, given the high availability of, and exposure to, high-calorie foods, decision-making has become a crucial factor in maintaining healthy eating habits. Since risk-taking is more prevalent in adolescence, it may be important to empower adolescents to make healthy decisions to prevent future obesity. Impulse control and decision-making should be an important target to prevent risky eating behaviour in adolescents.

## References

[1] Lee H, Lee D, Guo G, Harris KM. Trends in body mass index in adolescence and young adulthood in the United States: 1959–2002. J Adolesc Health. 2011;49: 601–608. 10.1016/j.jadohealth.2011.04.019 22098770 PMC3228354

[2] Ogden CL, Carroll MD, Kit BK, Flegal KM. Prevalence of obesity and trends in body mass index among US children and adolescents, 1999–2010. JAMA. 2012;12: 483–490. 10.1001/jama.2012.40 PMC6362452 22253364

[3] Singh AS, Mulder C, Twisk JWR, van Mechelen W, Chinapaw MJM. Tracking of childhood overweight into adulthood: a systematic review of the literature. Obes Rev. 2008;9: 474–488. 10.1111/j.1467-789X.2008.00475.x 18331423

[4] WHO: Obesity and overweight [Internet]. World Health Organization, 2018 Available from: http://www.who.int/news-room/fact-sheets/detail/obesity-and-overweight

[5] Zheng H, Lenard NR, Shin AC, Berthoud HR. Appetite control and energy balance regulation in the modern world: reward-driven brain overrides repletion signals. Int J Obes. 2009;33: 8–13. 10.1038/ijo.2009.65 PMC2838178 19528982

[6] Volkow ND, Wang GJ, Tomasi D, Baler RD. Obesity and addiction: neurobiological overlaps. Obes Rev. 2013;14: 2–18. 10.1111/j.1467-789X.2012.01031.x 23016694 PMC4827343

[7] Volkow ND, Wang GJ, Fowler JS, Tomasi D, Baler R. Food and drug reward: overlapping circuits in human obesity and addiction. Brain Imaging in Behav Neurosci. 2011;11: 1–24 10.1007/7854_2011_169 22016109

[8] Delgado-Rico E, Río-Valle JS, Gonzalez-Jiménez E, Campoy C, Verdejo-García A. BMI predicts emotion-driven impulsivity and cognitive inflexibility in adolescents with excess weight. Obesity. 2012;20: 1604–1610. 10.1038/oby.2012.47 22421897

[9] Ernst M, Fudge JL. A developmental neurobiological model of motivated behavior: anatomy, connectivity and ontogeny of the triadic nodes. ‎Neurosci Biobehav Rev. 2009;33: 367–382. 10.1016/j.neubiorev.2008.10.009 19028521 PMC2696617

[10] MacPherson L, Magidson JF, Reynolds EK, Kahler CW, Lejuez CW. Changes in sensation seeking and risk‐taking propensity predict increases in alcohol use among early adolescents. Alcohol Clin Exp Res. 2010;34: 1400–1408. 10.1111/j.1530-0277.2010.01223.x 20491737 PMC3123723

[11] Koritzky G, Yechiam E, Bukay I, Milman U. Obesity and risk taking. A male phenomenon. Appetite. 2012;59: 289–297. 10.1016/j.appet.2012.05.020 22634199

[12] Lane SD, Cherek DR. Analysis of risk taking in adults with a history of high risk behavior. Drug Alcohol Depend. 2000;60: 179–187. 10940545 10.1016/s0376-8716(99)00155-6

[13] Ball SA, Carroll KM, Rounsaville BJ. Sensation seeking, substance abuse, and psychopathology in treatment-seeking and community cocaine abusers. J Consult Clin Psychol. 1994;62: 1053–1057. 7806714 10.1037//0022-006x.62.5.1053

[14] Yechiam E, Busemeyer JR, Stout JC, Bechara A. Using cognitive models to map relations between neuropsychological disorders and human decision making deficits. Psychol Sci. 2005;16: 973–978. 10.1111/j.1467-9280.2005.01646.x 16313662

[15] Brogan A, Hevey D, O’Callaghan G, Yoder R, O’Shea D. Impaired decision making among morbidly obese adults. J Psychosom Res. 2011;70: 89–196. 10.1016/j.jpsychores.2010.07.012 21262422

[16] Arnett J. Reckless behavior in adolescence: A developmental perspective. Dev Rev. 1992;12(4): 339–373.

[17] Steinberg L. Risk taking in adolescence: New perspectives from brain and behavioral science. Curr Dir Psychol Sci. 2007;16(2): 55–59.

[18] Loeber S, Grosshans M, Herpertz S, Kiefer F, Herpertz SC. Hunger modulates behavioral disinhibition and attention allocation to food-associated cues in normal-weight controls. Appetite. 2013;71: 32–39. 10.1016/j.appet.2013.07.008 23899903

[19] Volkow ND, Baler RD. NOW vs LATER brain circuits: implications for obesity and addiction. Trends Neurosci. 2015;38: 345–52. 10.1016/j.tins.2015.04.002 25959611

[20] Werthmann J, Field M, Roefs A, Nederkoorn C, Jansen A. Attention bias for chocolate increases chocolate consumption—an attention bias modification study. J Behav Ther Exp Psychiatry. 2014;45: 136–143. 10.1016/j.jbtep.2013.09.009 24140811

[21] Doolan KJ, Breslin G, Hanna D, Gallagher AM. Attentional bias to food-related visual cues: Is there a role in obesity? Proc Nutr Soc. 2014;74: 37–45. 10.1017/S002966511400144X 25236786

[22] Nijs IMT, Muris P, Euser AS, Franken IHA. Differences in attention to food and food intake between overweight/ obese and normal-weight females under conditions of hunger and satiety. Appetite. 2010;54: 243–254. 10.1016/j.appet.2009.11.004 19922752

[23] Batterink L, Yokum S, Stice E. Body mass correlates inversely with inhibitory control in response to food among adolescent girls: an fMRI study. Neuroimage. 2010;52: 1696–1703. 10.1016/j.neuroimage.2010.05.059 20510377 PMC2910204

[24] Werthmann J, Jansen A, Roefs A. Worry or craving? A selective review of evidence for food-related attention biases in obese individuals, eating-disorder patients, restrained eaters and healthy samples. Proc Nutr Soc. 2015;74: 99–114. 10.1017/S0029665114001451 25311212

[25] Field M, Cox WM. Attentional bias in addictive behaviors: A review of its development, causes, and consequences. Drug Alcohol Depend. 2008;97: 1–20. 10.1016/j.drugalcdep.2008.03.030 18479844

[26] Jones JD, Vadhan NP, Luba RR, Comer SD. The effects of heroin administration and drug cues on impulsivity. J Clin Exp Neuropsychol. 2016;38: 709–720. 10.1080/13803395.2016.1156652 27062912 PMC4981922

[27] Lejuez CW, Aklin WM, Zvolensky MJ, Pedulla CM. Evaluation of the Balloon Analogue Risk Task (BART) as a predictor of adolescent real-world risk-taking behaviours. J Adolesc. 2003;26: 475–479. 12887935 10.1016/s0140-1971(03)00036-8

[28] Cole TJ, Lobstein T. Extended international (IOTF) body mass index cut‐offs for thinness, overweight and obesity. ‎Pediatr Obes. 2012;7: 284–294. 10.1111/j.2047-6310.2012.00064.x 22715120

[29] Cándido A, Orduña E, Perales JC, Verdejo-García A, Billieux J. Validation of a short Spanish version of the UPPS-P impulsive behaviour scale. Trastor Adict. 2012;14: 73–78.

[30] Lejuez CW, Read JP, Kahler CW, Richards JB, Ramsey SE, Stuart GL, et al Evaluation of a behavioral measure of risk taking: the Balloon Analogue Risk Task (BART). ‎J Exp Psychol Appl. 2002;8: 75 12075692 10.1037//1076-898x.8.2.75

[31] Preacher KJ, Hayes AF. SPSS and SAS procedures for estimating indirect effects in simple mediation models. Behav Res Meth Instrum Comput. 2004;36: 717–731. 10.3758/bf03206553 15641418

[32] Preacher KJ, Hayes AF. Asymptotic and resampling strategies for assessing and comparing indirect effects in multiple mediator models. Behav Res Meth. 2008;40: 879–891. 10.3758/brm.40.3.879 18697684

[33] Rothman KJ. No adjustments are needed for multiple comparisons. Epidemiol. 1990;43–46. 2081237

[34] Rothman KJ. Six Persistent Research Misconceptions. J Gen Intern Med. 2014;29: 1060–1064. 10.1007/s11606-013-2755-z 24452418 PMC4061362

[35] Yeomans MR, Brace A. Cued to act on impulse: more impulsive choice and risky decision making by women susceptible to overeating after exposure to food stimuli. PloS one. 2015;10: e0137626 10.1371/journal.pone.0137626 26378459 PMC4574976

[36] Stunkard AJ, Messick S. The three-factor eating questionnaire to measure dietary restraint, disinhibition and hunger. J Psychosom Res. 1985;29: 71–83. 3981480 10.1016/0022-3999(85)90010-8

[37] Yuen KSL, Lee TMC. Could mood state affect risk-taking decisions? J Affect Disord. 2003;75: 11–18. 12781345 10.1016/s0165-0327(02)00022-8

[38] Del Boca FK, Darkes J, Greenbaum PE, Goldman MS. Up close and personal: Temporal variability in the drinking of individual college students during their first year. J Consult Clin Psychol. 2004;72: 155–164. 10.1037/0022-006X.72.2.155 15065951

[39] Cooper ML, Agocha VB, Sheldon MS. A motivational perspective on risky behaviors: The role of personality and affect regulatory processes. J Pers. 2000;68: 1059–1088. 11130732 10.1111/1467-6494.00126

[40] Fernández-Serrano MJ, Moreno-López L, Pérez-García M, Viedma-del Jesús MI, Sánchez-Barrera MB, Verdejo-García A. Negative mood induction normalizes decision making in male cocaine dependent individuals. Psychopharmacology. 2011;217: 331–339. 10.1007/s00213-011-2288-2 21484236

[41] Stoeckel LE, Weller RE, Cook EW, Twieg DB, Knowlton RC, Cox JE. Widespread reward-system activation in obese women in response to pictures of high-calorie foods. Neuroimage. 2008;41: 636–647. 10.1016/j.neuroimage.2008.02.031 18413289

[42] Gray JA. The psychology of fear and stress. Cambridge: Cambridge University Press; 1987.

[43] Moreno-López L, Soriano-Mas C, Delgado-Rico E, Rio-Valle JS, Verdejo-García A. Brain structural correlates of reward sensitivity and impulsivity in adolescents with normal and excess weight. PloS one. 2012;7: e49185 10.1371/journal.pone.0049185 23185306 PMC3504042

[44] Robbins RN, Bryan A. Relationships between future orientation, impulsive sensation seeking, and risk behavior among adjudicated adolescents. J Adolesc Res. 2004;19: 428–445. 10.1177/0743558403258860 16429605 PMC1317100

[45] Nazarboland N, Fath N. The Role of BMI in Predicting Emotion-Driven Impulsivity and Sensitivity to Reward/Punishment in Over-Obese Adolescents. Biomed Pharmacol J. 2015;8: 729–737.

[46] Zuckerman M. Sensation seeking and the endogenous deficit theory of drug abuse. NIDA Res Monogr 1986;74: 59–70. 3122054

[47] Davis C, Patte K, Levitan R, Reid C, Tweed S, Curtis C. From motivation to behaviour: A model of reward sensitivity, overeating, and food preferences in the risk profile for obesity. Appetite. 2007;48: 12–19. 10.1016/j.appet.2006.05.016 16875757

[48] Nederkoorn C, Houben K, Hofmann W, Roefs A, Jansen A. Control yourself or just eat what you like? Weight gain over a year is predicted by an interactive effect of response inhibition and implicit preference for snack foods. Health Psychol. 2010;29: 389 10.1037/a0019921 20658826

[49] Velázquez-Sánchez C, Ferragud A, Moore CF, Everitt BJ, Sabino V, Cottone P. High trait impulsivity predicts food addiction-like behavior in the rat. Neuropsychopharmacology. 2014;39: 2463–2472. 10.1038/npp.2014.98 24776685 PMC4138758

[50] Epstein LH, Leddy JJ. Food reinforcement. Appetite. 2006;46: 22–25. 10.1016/j.appet.2005.04.006 16257474

[51] Berridge KC. ‘Liking’and ‘wanting’food rewards: brain substrates and roles in eating disorders. ‎Physiol Behav. 2009;97: 537–550. 10.1016/j.physbeh.2009.02.044 19336238 PMC2717031

[52] Millar L, Rowland B, Nichols M, Swinburn B, Bennett C, Skouteris H, et al Relationship between raised BMI and sugar sweetened beverage and high fat food consumption among children. Obesity. 2014;22: 96–103. 10.1002/oby.20665 24318968

[53] Macchi R, MacKew L, Davis C. Is decision-making ability related to food choice and facets of eating behaviour in adolescents? Appetite. 2017;116: 442–455. 10.1016/j.appet.2017.05.031 28536057

[54] Lejuez CW, Aklin WM, Bornovalova MA, Moolchan ET. Differences in risk-taking propensity across inner-city adolescent ever-and never-smokers. Nicotine Tob Res. 2005;7: 71–79. 10.1080/14622200412331328484 15804679

[55] Cosenza M, Griffiths MD, Nigro G, Ciccarelli M. Risk-taking, delay discounting, and time perspective in adolescent gamblers: An experimental study. J Gambl Stud. 2017;33: 383–395. 10.1007/s10899-016-9623-9 27256371

[56] Hanson KL, Thayer RE, Tapert SF. Adolescent marijuana users have elevated risk-taking on the balloon analog risk task. J Psychopharmacol. 2014;28: 1080–1087. 10.1177/0269881114550352 25237125 PMC8898087

